# SNP in human ARHGEF3 promoter is associated with DNase hypersensitivity, transcript level and platelet function, and Arhgef3 KO mice have increased mean platelet volume

**DOI:** 10.1371/journal.pone.0178095

**Published:** 2017-05-23

**Authors:** Siying Zou, Alexandra M. Teixeira, Myrto Kostadima, William J. Astle, Aparna Radhakrishnan, Lukas Mikolaj Simon, Lucy Truman, Jennifer S. Fang, John Hwa, Ping-xia Zhang, Pim van der Harst, Paul F. Bray, Willem H. Ouwehand, Mattia Frontini, Diane S. Krause

**Affiliations:** 1 Department of Cell Biology, Yale School of Medicine, New Haven, Connecticut, United States of America; 2 Department of Pathology, Yale School of Medicine, New Haven, Connecticut, United States of America; 3 Department of Haematology, University of Cambridge, Cambridge Biomedical Campus, Cambridge, United Kingdom; 4 Department of Structural and Computational Biology, Baylor College of Medicine, Houston, TX, United States of America; 5 Department of ENT, Addenbrooke’s Hospital, Cambridge Biomedical Campus, Cambridge, United Kingdom; 6 Yale Cardiovascular Research Center, Department of Cardiology, Yale University School of Medicine, New Haven, CT, United States of America; 7 Department of Laboratory Medicine, Yale School of Medicine, New Haven, Connecticut, United States of America; 8 Yale Stem Cell Center, Yale School of Medicine, New Haven, Connecticut, United States of America; 9 University of Groningen, University Medical Center Groningen, Department of Cardiology, Groningen, The Netherlands; 10 University of Groningen, University Medical Center Groningen, Department of Genetics, Groningen, The Netherlands; 11 Department of Medicine and the Program in Molecular Medicine, University of Utah, Salt Lake City, Utah, United States of America; Ludwig-Maximilians-Universitat Munchen, GERMANY

## Abstract

Genome-wide association studies have identified a genetic variant at 3p14.3 (SNP rs1354034) that strongly associates with platelet number and mean platelet volume in humans. While originally proposed to be intronic, analysis of mRNA expression in primary human hematopoietic subpopulations reveals that this SNP is located directly upstream of the predominantly expressed *ARHGEF3* isoform in megakaryocytes (MK). We found that *ARHGEF3*, which encodes a Rho guanine exchange factor, is dramatically upregulated during both human and murine MK maturation. We show that the SNP (rs1354034) is located in a DNase I hypersensitive region in human MKs and is an expression quantitative locus (eQTL) associated with *ARHGEF3* expression level in human platelets, suggesting that it may be the causal SNP that accounts for the variations observed in human platelet traits and ARHGEF3 expression. In vitro human platelet activation assays revealed that rs1354034 is highly correlated with human platelet activation by ADP. In order to test whether ARHGEF3 plays a role in MK development and/or platelet function, we developed an *Arhgef3* KO/LacZ reporter mouse model. Reflecting changes in gene expression, LacZ expression increases during MK maturation in these mice. Although *Arhgef3* KO mice have significantly larger platelets, loss of *Arhgef3* does not affect baseline MK or platelets nor does it affect platelet function or platelet recovery in response to antibody-mediated platelet depletion compared to littermate controls. In summary, our data suggest that modulation of ARHGEF3 gene expression in humans with a promoter-localized SNP plays a role in human MKs and human platelet function—a finding resulting from the biological follow-up of human genetic studies. *Arhgef3* KO mice partially recapitulate the human phenotype.

## Introduction

Genome-wide association studies (GWAS) identify genomic polymorphisms associated with specific phenotypes and diseases in humans.[1–3] The description of these loci holds promise for the identification of important biological pathways and disease mechanisms. GWAS studies in humans have identified hundreds of single nucleotide polymorphisms (SNPs) associated with the number of platelets per unit blood volume (platelet count, PLT) and platelet size (mean platelet volume, MPV), which show natural variation in humans and are heritable traits. [4–8] One of the SNPs showing the strongest association with PLT and MPV in humans resides in the in the locus for the guanidine exchange factor ARHGEF3. The GWAS association signal indicated a potential functional role of this GEF protein in megakaryopoiesis and the formation and function of platelets. To assess for a functional role of ARHGEF3, we generated Arhgef3 knock-out mice. In order to further examine the potential functional outcome in human platelets, we analyzed the experimental results from the BLUEPRINT epigenome study and from the Cambridge Platelet Function cohort and the PRAX1 expression QTL study.

*ARHGEF3* encodes for the Rho Guanine Nucleotide Exchange Factor 3, also known as the Exchange Factor Found in Platelets, Leukemic, and Neuronal tissues (XPLN).[9] It regulates the switch of Rho GTPase from the inactive GDP-bound state to the active GTP-bound state, and is one of the most abundant GEFs found in human MK lineage [7, 8] and platelets.[9] It has also been implicated as playing a role in myeloid differentiation.[10] Published microarray data and western blot analyses confirm that ARHGEF3 is highly expressed in platelets.[9, 11] However, whether ARHGEF3 plays a role in megakaryocyte development, platelet formation and/or platelet function in mammals has not been reported.

Here, we used insights from human genetics to study the function of ARHGEF3 in primary murine and human megakaryocytes and platelets. We created an *Arhgef3* knockout (KO) mouse model with interruption of the endogenous gene by insertion of LacZ reporter cDNA. We found that *Arhgef3* KO mice have normal MK maturation and platelet function. However, KO mice have enlarged platelets and a mild delay in platelet recovery in response to thrombocytopenia. In the human system, we found that the rs1354034 SNP of *ARHGEF3* is an expression quantitative trait locus (eQTL) that strongly correlates with ADP induced fibrinogen binding of platelets *in vitro*. In addition, we show that rs1354034 may perturb function by disrupting protein binding to DNA in human MKs. Here we present evidence suggesting that ARHGEF3 plays an important role in human megakaryocytes and platelets. However, ARHGEF3 is less critical in murine megakaryocytes and platelets.

## Results

### ARHGEF3 is upregulated during MK differentiation

**Fig 1:**
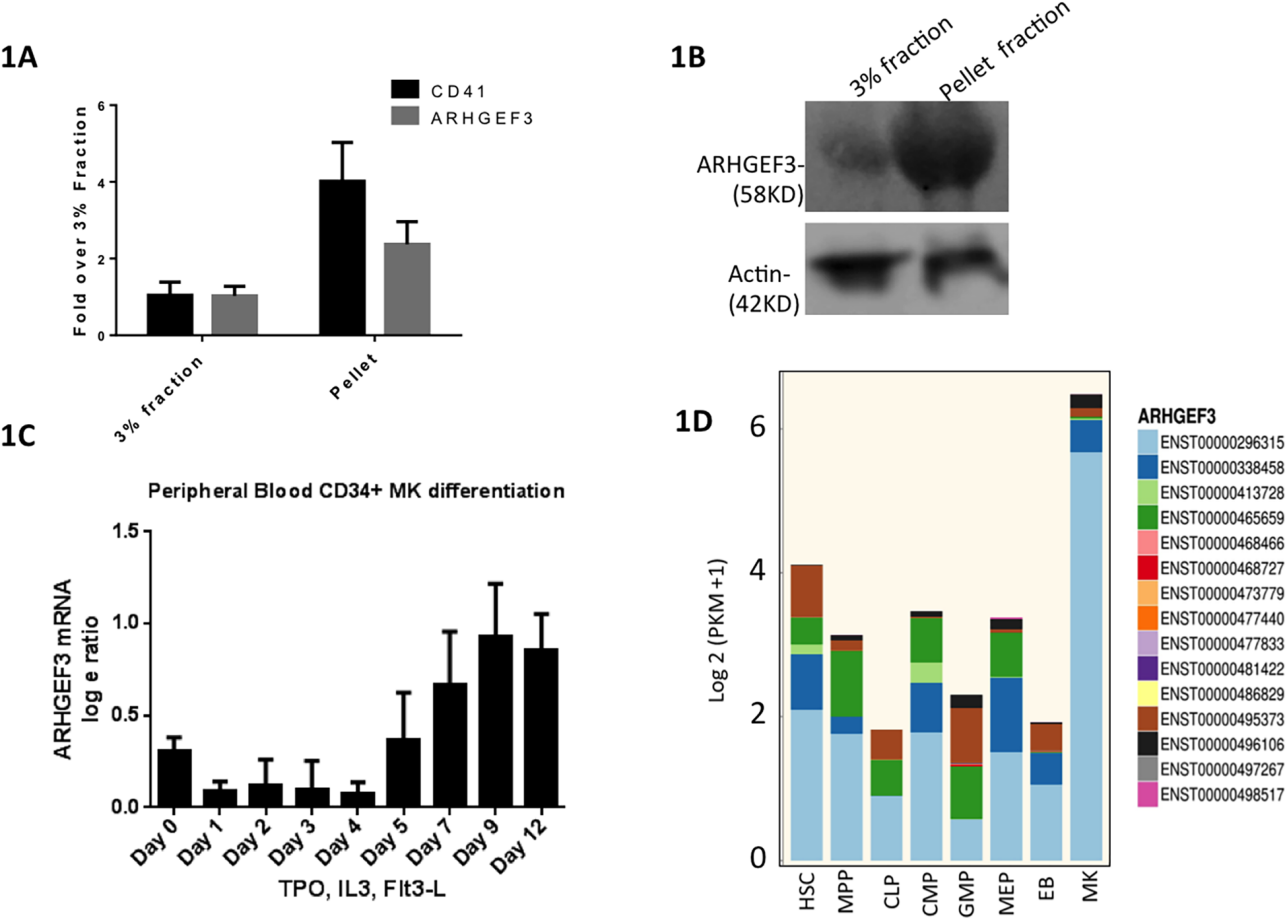
ARHGEF3 expression in primary murine and human cells. (A-B) ARHGEF3 mRNA and protein levels were assessed in megakaryocytes derived in vitro from WT E13.5 fetal liver cells. A 3% BSA gradient was used to separate megakaryocytes (MKs). Both CD41 (positive control) and ARHGEF3 expression are increased in the pellet fractions compared to the 3% BSA fraction (p < 0.005). (C) RNA sequencing data from public a database [12] shows ARHGEF3 upregulation during human CD34+ differentiation down the MK lineage. (D) Barplots representing transcript isoform expression levels across eight human hematopoietic cell types.[13] ARHGEF3 expression (represented by the total height of the barplots) increases during megakaryocytic lineage differentiation and the most abundant transcript isoform in megakaryocytes is ENST00000296315. The figure includes Ensembl v70 annotated transcripts and those annotated as non-protein coding or nonsense mediated decay have been grouped together. [HSC: hematopoietic stem cell, MPP: multipotent progenitor, CLP: common lymphoid progenitor, CMP: common myeloid progenitor, GMP: granulocyte monocyte progenitor, MEP: megakaryocyte erythrocyte progenitor, EB: erythroblasts and MK: megakaryocytes.]

To study the function of ARHGEF3 in mammalian megakaryocytes and platelets, we first determined the expression level of ARHGEF3 during megakaryocyte development. By Quantitative-RT-PCR (qRT-PCR) and western blot analysis, both ARHGEF3 RNA and protein were highly enriched in the pellet fraction (most highly enriched for mature MKs as shown by the CD41 level) compared to the 3% fraction of the differentiated fetal liver cells (p<0.005), suggesting that mature megakaryocytes express high ARHGEF3 (Fig 1A and 1B). We further studied human cells, where published microarray data on CD34-derived cells that had been differentiated in vitro down the MK lineage showed that *ARHGEF3* mRNA levels increased during MK maturation (Fig 1C).[12] RNA sequencing on eight human hematopoietic progenitor populations, performed by the BLUEPRINT consortium, revealed the highest levels of *ARHGEF3* expression in MK. The predominant isoform in MK (light blue, ENST00000296315, Fig 1D) is the second longest annotated transcript with a transcription start site in exon 5 of the longest transcript.[13] ARHGEF3 transcript 001 (ENST00000296315) has a posterior probability of being MK-specific of 0.996470891 with an estimated log fold change (relative all other cell types) of 2.66. [13]

### SNP rs1354034 is a leading SNP associated with *ARHGEF3* expression level

**Fig 2:**
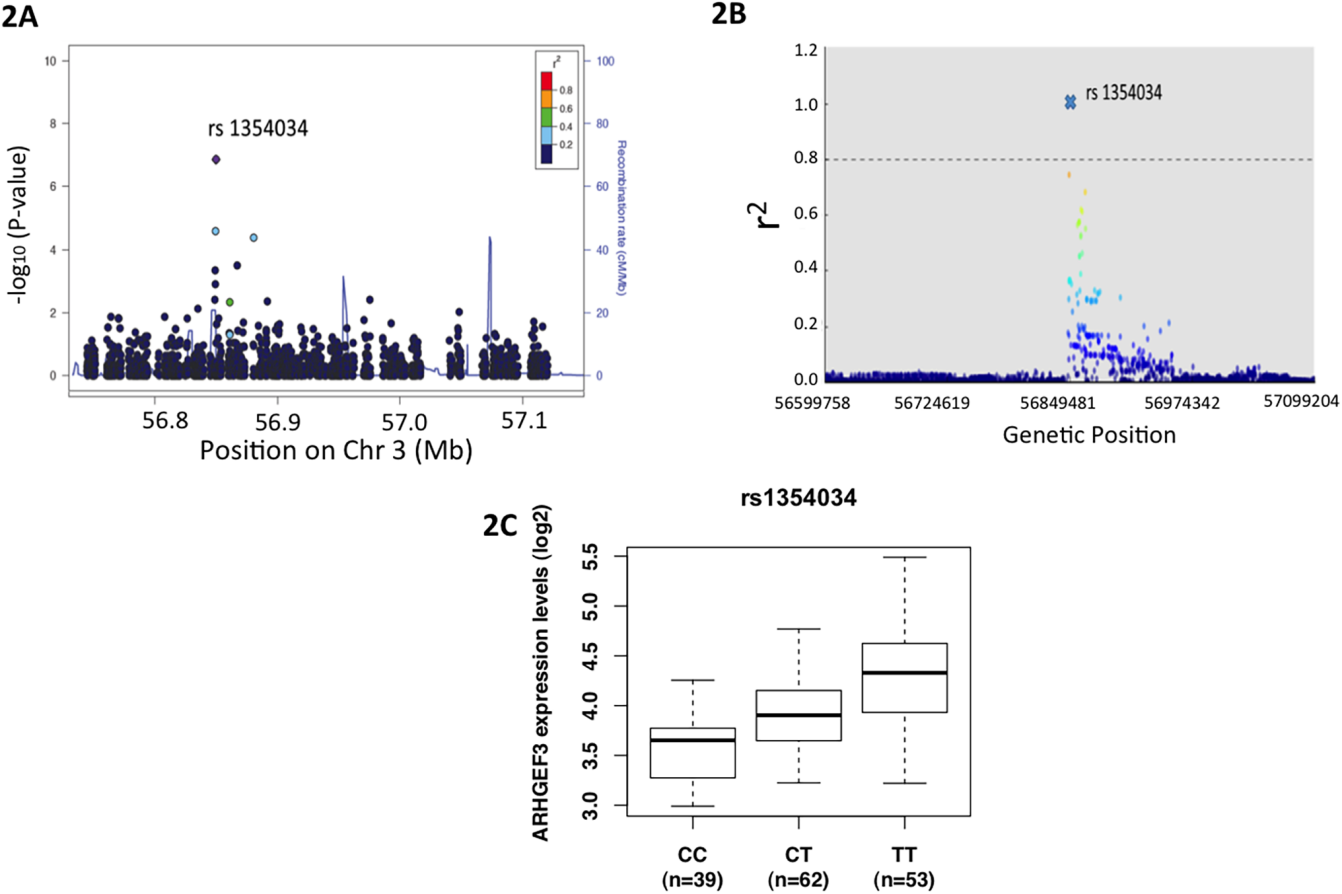
rs1354034 is the leading SNP and an eQTL for ARHGEF3 expression. (A) Visual summary of the statistical evidence for variant-phenotype association at the locus of rs1354034 from data generated by the TEMPLE targeted sequencing study. It shows the variant specific P-values from Fisher’s exact tests against null hypotheses that genotype frequencies are the same in the high and low platelet count samples. The color of each point represents the *r*^2^ between the corresponding variant and rs1354034, estimated from the TEMPLE genotypes. The continuous line indicates the local recombination rate estimated from HapMap data. (The tool used to generate the plot is available at doi:10.1093/bioinformatics/btq41.) (B) Shown is rs 1354034 (blue X) and its LD structure in +/- 250 kb window. There is no SNP in linkage disequilibrium (threshold *r*^2^ >0.8) found 250k bp up- or downstream of the rs1354034 locus (data from 1000 Genome Project), suggesting rs1354034 is the leading SNP of ARHGEF3. (C) A strong association of the rs1354034 genotype with ARHGEF3 mRNA levels (p = 1.37x10^-15^, ordinary linear regression). This association stayed significant even after accounting for subject age, gender and race (p = 2.39 x 10^−11^, multiple linear regression). Primary data for 5C in online S1 Table.

To further address whether ARHGEF3 plays a role in human MK and platelets, we confirmed the association between platelet count and genetic variation at the SNP (rs1354034) upstream of the *ARHGEF3* gene. A 400kb region containing *ARHGEF3* gene was resequenced in 500 people with sex-adjusted low platelet counts and 500 people with sex-adjusted high platelet counts. These healthy subjects represent the 5% tails of the age adjusted platelet distribution in the Dutch *LifeLines Cohort Study [14]* (for which platelet count had been measured in 94,753 study subjects). The SNP rs1354034 had the lowest *p*-value (*p* = 7.10×10^−6^) in a one variant at a time analysis testing for association between platelet distribution tail status (high/low) and genotype using Fisher’s exact test (Fig 2A), with 56%, 52% and 32% of CC, CT and TT genotypes respectively corresponding to study subjects in the upper 5% tail of platelet count (data not shown), thus the C-allele is associated with higher platelet counts. The association between the common SNP rs1354034 and the traits of mean volume and platelet count (PLT) has been replicated in two further GWAS. [7, 8] The results of these four independent studies render a false-positive association unlikely. Moreover, a closer examination of the nearby genomic region of the target SNP rs1354034 revealed that no other SNPs within 250k bp up/downstream of the rs1354034 locus in high linkage disequilibrium (threshold r^2^ = 0.8, Fig 2B) suggesting that rs1354034 is the leading SNP responsible for the human platelet variations observed.

Epigenetic analysis of the chromosome landscape of primary human MKs derived from CD34+ cord blood further revealed that rs1354034 resides about 15k bp upstream of the active promoter (at a H3K27 acetylation peak) of the predominant isoform of *ARHGEF3* identified by RNA-seq. This further corroborated that *ARHGEF3* transcript ENST00000296315 is expressed in MKs, and suggested that the SNP may perturb regulation of the expression of the *ARHGEF3* gene. We therefore assessed the association between *ARHGEF3* haplotype and mRNA levels by comparing the transcriptomes of platelets from individuals of different genotypes. Data from the PRAX1 study on 154 healthy human subjects [11] showed that rs1354034 is strongly associated (p<0.001) with *ARHGEF3* expression in platelets (Fig 2C). Further analysis within the population showed that the allele frequencies of SNP rs1354034 are differentially distributed across different ethnicities in the human population (S1 Fig). The fact that the same haplotype associates with hematological platelet parameters and *ARHGEF3* expression provides strong evidence that the differences in platelet count and volume observed between genotype groups are mechanistically related to differences in *ARHGEF3* levels.

### SNP rs1354034 is associated with human platelet function

**Fig 3:**
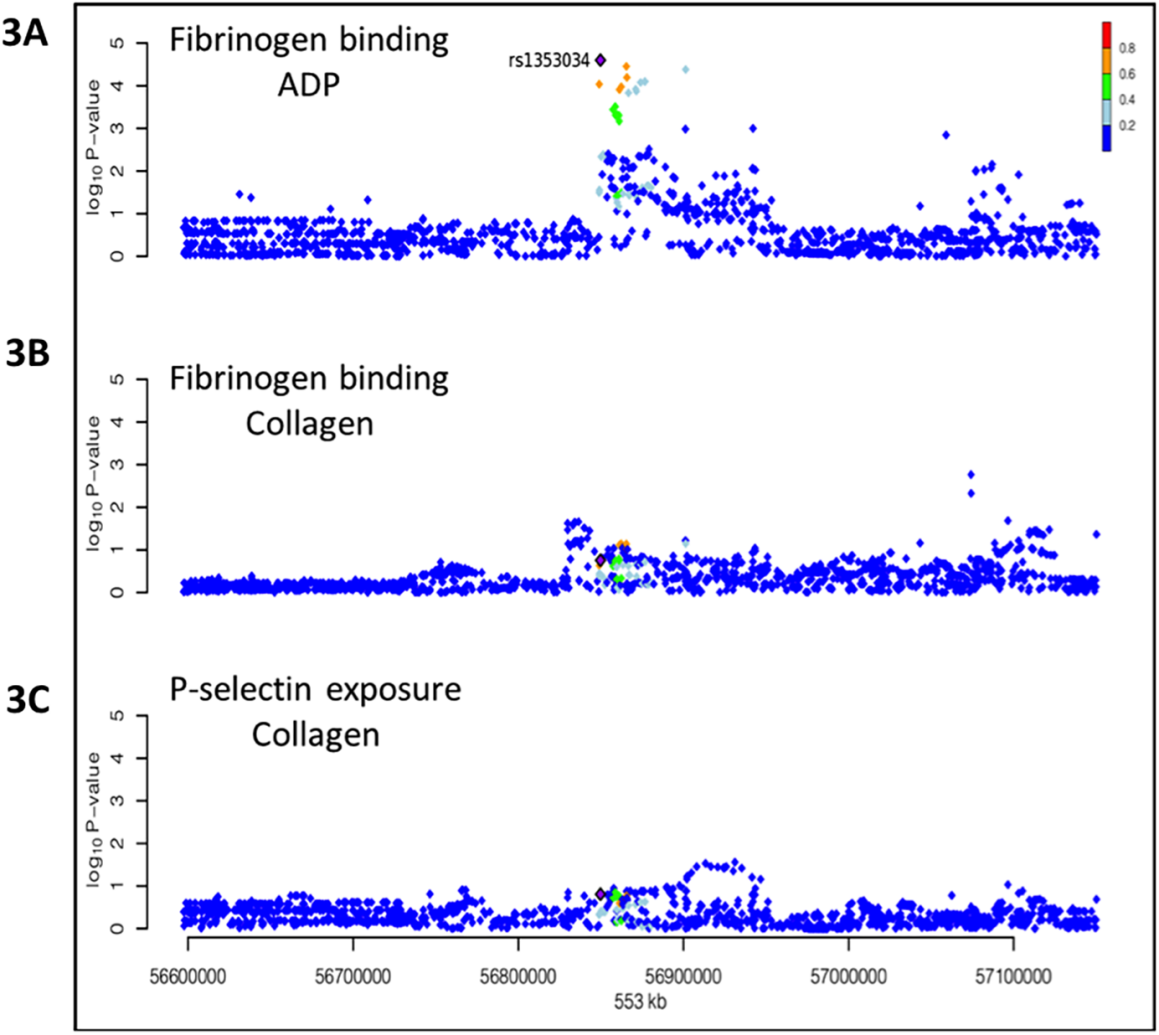
rs1354034 is associated with human platelet function. (A-C) Association studies on SNPs and human platelet function. X axis: Genomic position. Y axis: -log10 (P-value from linear additive association). The three quantitative traits measured by flow cytometry are fibrinogen binding in response to ADP (A) or Collagen (B) and P-selectin expression in response to Collagen (C). The purple diamond represents the lead SNP and the colour of the other SNPs is based on the linkage disequilibrium (in terms of *r*^*2*^) with the lead SNP.

The positive correlation between platelet volume and functional activity has been hypothesized and explored in platelet biology.[15] We further performed association studies on the platelet count and volume loci identified in the GWA studies with the human platelet functional response to ADP and collagen. Specifically, we tested in the Cambridge platelet function cohort of just more than 1,200 genome-wide typed individuals, the association between allelic variants and two functional read-outs by flow cytometry: 1) fibrinogen binding and 2) P selectin expression levels. Platelets were activated with either the collagen mimetic CRP-XL or ADP. A total of 39,331,925 variants obtained after imputation of the genotypes using the results of the 1000 Genomes project for reference were tested for association with the four traits. Four genetically independent variants were identified as being associated with at least one of the four platelet function traits at P< 5x10^-7^, with rs1354034 in ARHGEF3 being one of the four (P values: 6.9 x 10^−8^ and 5.5 x 10^−8^ for fibrinogen and P-selectin, respectively). The levels of functional response are lower in subjects carrying the minor (C) allele of rs1354034. It is interesting to note that no association signal was observed for these two markers after activation of platelets by collagen (Fig 3), indicating a highly specific action of ARHGEF3 on signaling events downstream of the G-protein coupled ADP receptors P2RY1 and P2RY12. Thus, rs1354034 is uniquely associated with fibrinogen binding in response to ADP stimulation.

### SNP rs1354034 resides in an open chromatin region and may affect binding of chromatin-associated proteins in MKs

**Fig 4:**
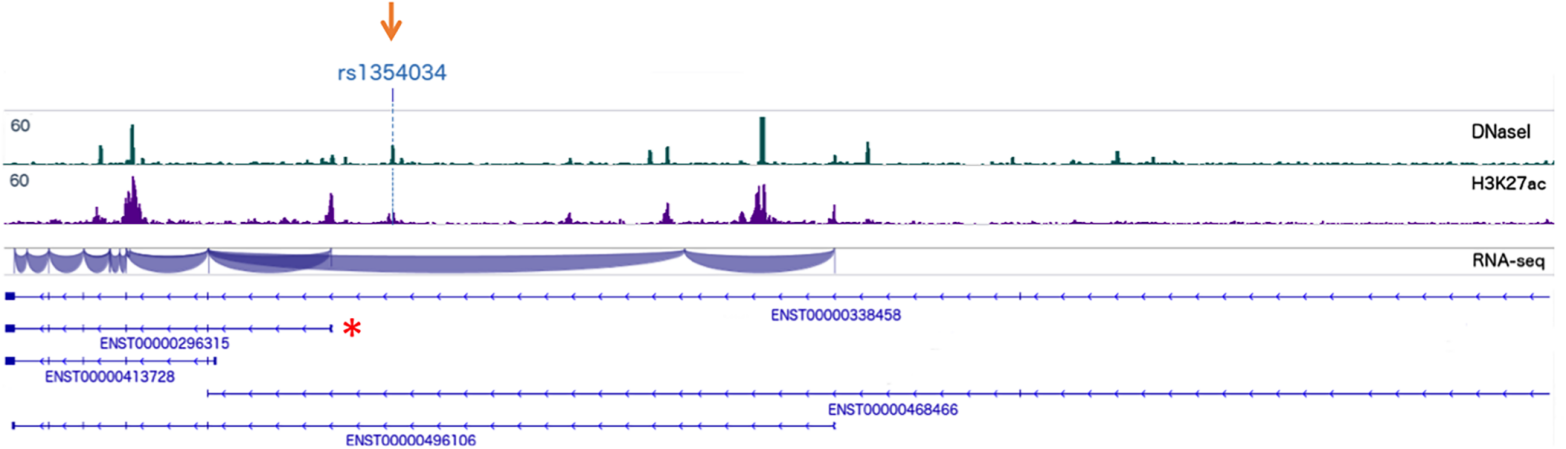
rs1354034 is located in a DNase I hypersensitive region in primary human MKs. Shown is the chromatin locus containing the ARHGEF3 gene. rs1354034 (orange arrow) is located in an open-chromatin (DNase I peak) non-coding region upstream of the transcription start site of ENST00000296315 (the most commonly transcribed mRNA in MK, indicated by an asterisk). Green: DNase 1 peaks, Purple: H3K27Ac ChIP peaks, Blue: intron/exon boundaries, Bottom: major ENST’s identified for ARHGEF3.

Because platelets do not have a nucleus, the finding that the genomic locus rs1354034 strongly associates with human platelet traits (volume, count and function) prompted us to study the locus in MK from a genomic and epigenetic perspective, using human MK derived *in vitro* from CD34+ cord blood. We found that the SNP rs1354034 is located in a DNase I hypersensitive site (Fig 4, green) that is also marked for H3K27ac histone modification (Fig 4, purple), suggesting that the SNP lies within a regulatory region and may alter protein binding to this genomic locus in MK. Examining chromatin immunoprecipitation followed by DNA sequencing (ChIP-seq) from the ENCODE project (http://genome.ucsc.edu/cgi-bin/hgTrackUi?db=hg19&g=wgEncodeRegTfbsClusteredV3) for transcription factors for MK maturation (SCL, RUNX1, MEIS1, GATA2, GATA1, and FLI1 [13, 16]), none of these factors was found to bind specifically at the SNP site.

### Production and validation of *Arhgef3* knockout (KO) mice

**Fig 5:**
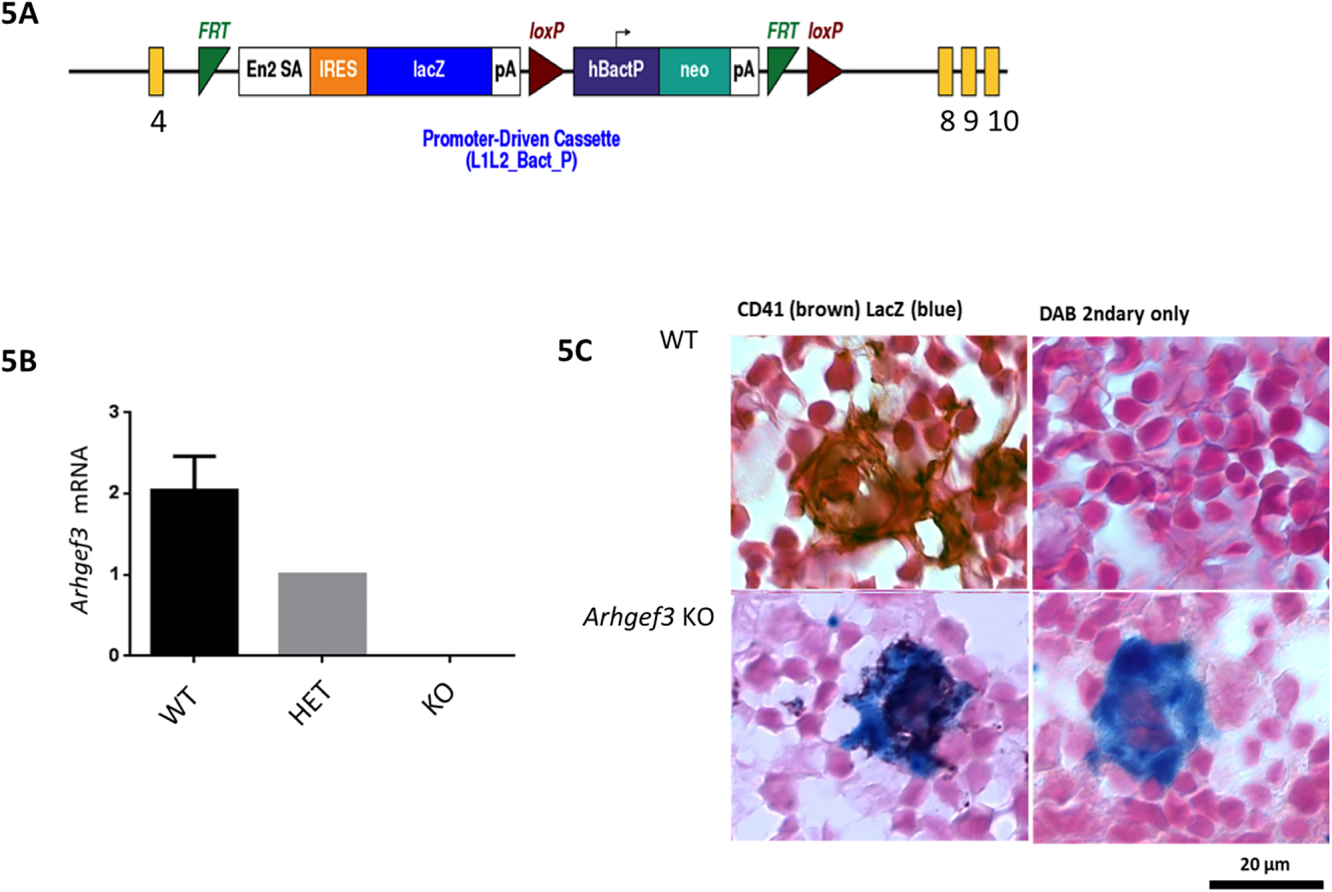
ARHGEF3 knockout design and confirmation of *Arhgef3* knockout in the megakaryocyte lineage. (A) *Arhgef3* KO design from KOMP: The wild type *Arhgef3* gene is disrupted by replacement of exons 5–7 with a gene cassette containing a splice acceptor (En2SA), an IRES and LacZ cDNA. The neomycin resistance gene has its own beta actin promoter. (B) *Arhgef3* KO confirmation: qRT-PCR for *Arhgef3* in fresh bone marrow cells from WT, ARHGEF3 heterozygous and homozygous KO mice confirmed the complete loss of ARHGEF3 in the KO mice. (C) LacZ/β-galactosidase staining on E13 mouse liver from wild type (WT) or *Arhgef3* homozygous knockout embryos shows that *Arhgef3* KO MKs are positive for both CD41 (brown) and LacZ/β-galactosidase staining (blue).

We produced a global *Arhgef3* knockout (KO) mouse model with *Arhgef3* KO embryonic stem (ES) cells purchased from the Knockout Mouse Project Repository (KOMP) (Fig 5A). In these cells and the mice derived therefrom, the *Arhgef3* genomic locus is deleted from exon 5–7 and is replaced with a splice acceptor (En2SA) followed by an Internal Ribosome Entry Site (IRES), LacZ cDNA and a poly-adenine sequence (PA). This allows LacZ to serve as a reporter for expression of the endogenous *Arhgef3* gene.

The *Arhgef3* global KO mice obtained are fertile and viable, with no obvious developmental defects. qRT-PCR on freshly isolated bone marrow cells from WT, *Arhgef3* heterozygotes (HET) and homozygotes knockout (KO) mice confirmed that there is a complete loss of *Arhgef3* mRNA in the KO and a 50% decrease of mRNA level in the heterozygotes (Fig 5B). LacZ expression in the *Arhgef3* KO megakaryocytes (CD41+) was confirmed by immunohistochemistry for β-galactosidase and CD41+ (Fig 5C) on day E13.5 mouse embryo liver, consistent with high ARHGEF3 protein expression in MKs.

### *Arhgef3* KO mice have enlarged platelets, but normal platelet function

**Fig 6:**
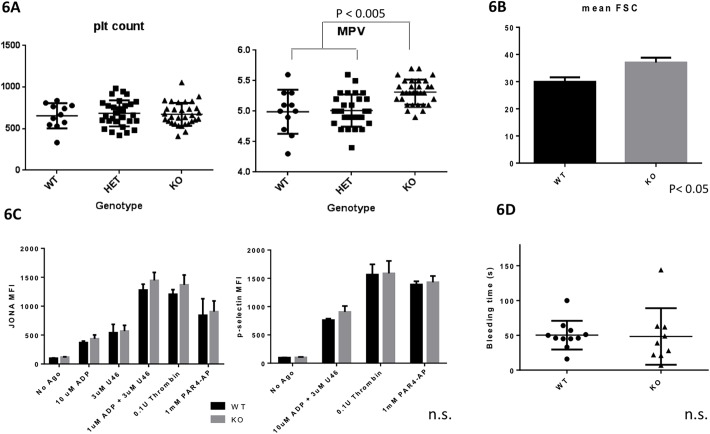
*Arhgef3* KO mice have enlarged platelets, and normal platelet activation. (A) Peripheral blood was taken from 4 week old littermate mice with the indicated genotypes. *Arhgef3* KO mice have significantly larger platelets (MPV) but normal platelet counts. (B) Mean forward side scatter (FSC) of platelets (gated as CD41/CD61+ from whole blood) confirmed that KO mice have larger platelets. (C) Flow cytometry for Integrin α2bβ3 conformational change (JONA MFI) and P-selectin exposure of platelets from littermate mice in response to ADP, thromboxane (U46), thrombin and PAR4-AP stimulation. There are no significant differences between WT and *Arhgef3* KO platelets with any of the agonists tested. (3 independent experiments, n = 4/genotype). (D) Tail bleeding times of WT and *Arhgef3* KO littermates showed no differences in bleeding time. (WT n = 10, KO n = 9).

We found that there is a small, but statistically significant (p<0.005), increase in mean platelet volume in KO mice (KO: 5.313 ± 0.03702 fl, n = 31; WT: 4.991 ± 0.1091 fl, n = 11). But, platelet counts are indistinguishable between WT and KO animals (Fig 6A). We assayed *in vitro* platelet function by flow cytometry with the JONA antibody, which binds specifically to activated integrin α2bβ3 (inside-out signaling), and anti- P-selectin (CD62), which assesses granule release, in response to multiple agonists. In the resting state, *Arhgef3* KO platelets displayed higher forward scatter (FSC) than WT (Fig 6B), consistent with the increased platelet volume. However, there was no significant defect in the *Arhgef3* KO versus WT platelet in activation with any of the agonists tested (Fig 6C). We further tested *in vivo* platelet function by the tail bleeding time assay, and no differences were found in the KO compared to WT littermates (Fig 6D). Taken together, these results indicate that platelet function is normal in the absence of Arhgef3.

#### *Arhgef3* KO mice have normal MK maturation, but have delayed platelet recovery

We assessed platelet formation in the absence of ARHGEF3 by monitoring *in vivo* platelet recovery following antibody-mediated acute thrombocytopenia. There was no significant effect on time to platelet recovery in the KO mice (data not shown). We also assessed bone marrow MK maturation. Analysis of baseline (unstimulated) bone marrow by flow cytometry revealed no differences between KO and WT littermates in the percentages of different progenitor populations, including Lin−Sca+Kit+ stem/progenitor cells, CD41-CD150+CD105- megakaryocyte-erythroid progenitors (PreMegE) or CD41+Kit+ megakaryocyte progenitors (MKP) (S2A Fig). To further look into the function of these progenitor cells, we performed analysis for mature MKs on fresh or *in vitro* differentiated bone marrow cells from WT and *Arhgef3* KO mice. Again, no differences were found in maturation (co-expression of MK cell surface markers CD41 and CD42) or DNA ploidy in the bone marrow MK between WT and KO mice (S2B Fig). Thus, Arhgef3 is not critical for murine MK development.

## Discussion

In the present study, we performed follow-up research on one of the top GWA studies hits–rs1354034 proximal to the gene *ARHGEF3*, a little known RhoA guanine exchange factor–in both primary murine and human megakaryocytes and platelets. Although a relevant role of ARHGEF3 in platelets has been suggested from multiple recent GWA studies in human cohorts as well as by morpholino knockdown (KD) experiments in the zebrafish [4–6], the question of whether the manipulation of ARHGEF3 levels alters megakaryocyte or platelet function in the mouse system has not been assessed, nor is the biological mechanisms of ARHGEF3 function in human cells known. Here, we further analyzed the SNP of *ARHGEF3* in primary human megakaryocytes and platelets, and we created a global knockout mouse model of *Arhgef3* in which we analyzed the megakaryocyte and platelet phenotypes.

Several lines of evidence from the literature and our analyses suggest that ARHGEF3 may play a role in human megakaryocytes and platelets. 1) Our studies show that ARHGEF3 is upregulated during megakaryocytic differentiation and enriched in mature megakaryocytes in both mice and humans. 2) The SNP rs1354034 is the leading SNP for the variations of platelet count and volume. 3) The SNP is an eQTL associated with *ARHGEF3* expression level in platelets. 4) The same locus also associates with human platelet function unique to increased fibrinogen binding in response to ADP stimulation. 5) The SNP localizes to a DNase hypersensitive site in an open chromatin that also localizes H3K27Ac, and thus may regulate ARHGEF3 expression in human megakaryocytes. However, given the “negative” findings of this study, we cannot yet clarify the mechanism through which genetic variation in ARHGEF3 alters PLT and MPV in humans. Possiblities include: 1) the SNP affects a unique regulatory network in human cells that remains to be discovered, 2) ARHGEF3 plays a role in humans that is not shared in mice, or 3) ARHGEF3 does not effect megakaryocytes or platelets in humans, and the SNP is affecting something unrelated to the gene in which it is located.

ARHGEF3 deficient mice appear to have normal megakaryocyte development and platelet function, and restore their platelet counts to baseline similarly to WT mice in response to acute platelet depletion in vivo. Given that our data demonstrate that the C-allele in rs1354034 is associated with lower *ARHGEF3* mRNA expression, higher mean plt count and lower MPV in humans, we had predicted that *Arhgef3* KO would potentially lead to lower platelet counts and higher MPV in mice. Our findings, however, were that Arhgef3 KO had no effect on murine platelet counts and a statistically significant increase in the MPV. The mechanisms underlying this association are not yet clear.

The largely normal megakaryocyte and platelet function of the *Arhgef3* KO mice was surprising, but may be explained by differences in the zebrafish and humans versus mouse megakaryopoiesis and thrombopoiesis. The same research group that performed *Arhgef3* morpholino knockdown (KD) experiment in zebrafish found that in addition to the thrombocyte phenotype, *Arhgef3* KD also results in abrogation of erythroid maturation.[17] This suggests that ARHGEF3 may affect early stages of hematopoiesis in the fish. Although humans and mice are evolutionarily closer, there are of course many differences between the two species with potentially important biological and translational implications.[18] Thus, a more suitable model to test the ARHGEF3 SNP might be a transgenic mouse with the same SNP variations found in humans.

Another possible explanation for the mild phenotype of the *Arhgef3* KO is the existence of compensatory mechanisms related to other guanine exchange factors. The SNP in *ARHGEF3* was identified within a healthy cohort, where association of the gene expression level (the SNP is an eQTL) is identified with variations of platelet traits.[4–6] However, in a mouse system where the gene is completely knocked out, there may be compensatory mechanisms to prevent effects due to loss of a critical gene. There are over 80 different GEF proteins expressed in mammals spatially and temporarily regulating different cellular processes, and all of them target one of the three main substrate families—Rho, Rac, CDC42.[19] The most highly conserved GEF to ARHGEF3 is Neuroepithelial Cell Transforming 1 (NET1). When we knocked down *Net1* in *Arhgef3* KO murine cells, there was no effect on megakaryocyte maturation suggesting that Net1 does not compensate for Arhgef3 (data not shown).

Also, SNP association studies in different ethnicities reveal that the allele frequencies of SNP rs1354034 are differentially distributed across different ethnicities in the human population (S2 Fig). Previous research has found such ethnic variation in the human population can be reflected as strain variation in mouse models as well.[20] Hence, the inbred mouse strains used in research may also influence the observed phenotypes.

In this study, we further examined ARGHEF3 in human cells. We found that the *ARHGEF3* SNP strongly associates with ADP-stimulated fibrinogen binding but not collagen-stimulated fibrinogen binding or P-selectin exposure in human platelets. The two agonists activate two distinct signaling cascades leading to platelet activation. Collagen activates platelets via GPVI (GP6) and FcRγ/ITAM (FCER1G) signaling cascade [21, 22] while ADP amplifies the activation of platelets through its binding to the two G protein–coupled receptors, P2Y12 and P2Y1. P2Y12, through activating GTP-bound α subunit Gαi, suppresses cAMP formation so that platelet activation can occur. P2Y1, activates phospholipase C through GTP-bound α subunit Gαq, which moderates Calcium release.[23] Platelets from mice lacking P2Y1 do not undergo shape change in response to ADP,[24] suggesting that ADP signaling is involved in platelet shape change, mechanism of which is unclear. Given that platelet shape change is mediated through Rho signaling and actin reorganization,[25] it is reasonable to hypothesize that ARHGEF3 may be the missing link between ADP signaling through P2Y1 receptor and Rho-mediated shape change in human platelets. Interestingly, in another genome-wide association study, the same SNP, rs1354034 on chromosome 3, was found to be associated *in trans* with expression levels of von Willebrand Factor (vWF), an important blood coagulation gene on chromosome 12, with a P value of 5.43 × 10^−5^.[26] This further supports that rs1354034 correlates with human platelet function.

In summary, based on previously published GWA studies and the published morpholino knockdown experiments in the zebrafish, we tested the function of ARHGEF3 in both murine and human megakaryocytes and platelets. While there was no phenotype in the mice aside from an unexpected increase in MPV, our study suggests new hypotheses for further validation and studies of the molecular mechanism of the SNP locus in human MK development and the role of ARHGEF3 in human platelet activation, especially testing the hypothesis that ARHGEF3 is downstream of ADP stimulated P2Y1 and Gαq activation to mediate Rho activity for platelet shape change. Our study also proposes that transgenic mouse models with the same SNP variations, when possible, would be better models for mimicking gene variations in humans.

## Materials and methods

### Mice

ARHGEF3 knockout ES cells (https://www.komp.org/pdf.php?projectID=39618, derived from JM8A3.N1 ES cells, strain background: C57BL/6N-Atm1Brd, which is essentially Bl6 with the agouti allele) were purchased from Knockout Mouse Project Repository (KOMP). The ES cells were microinjected into albino B6 mice blastocysts using standard techniques.[27] The chimeric mice were generated and mated to obtain homozygous KO mice. These mice are now available from Jackson Laboratories (Stock No. 028838 C57BL/6N-Arhgef3<tm1(KOMP)Wtsi>/DkrsJ). For all experiments, wild-type (WT) littermates served as controls. All mouse procedures were performed according to Yale University Animal Care and Use Committee-approved protocols and complied with federal laws.

### Quantitative RT-PCR

RNA from 5x10^4^ cells was isolated using the RNAqueous-Micro Kit (Life Technology, Carlsbad, CA), and treated with RNase-free DNase I. First-strand cDNA was produced with iScript™ cDNA Synthesis Kit (Bio-Rad, Hercules, CA) with 20 ng RNA from each sample. Gene expression levels were quantified on an iCycler iQ RT machine (Bio-Rad, Hercules, CA, USA) with 2 ul of cDNA product from each sample using TaqMan probes (Applied Biosystems, Carlsbad, CA) as follows: murine ARHGEF3: Mm00551104_m1 and Eukaryotic18S rRNA: Hs99999901_s1. Relative gene levels were calculated from standard curves and normalized to 18 s levels.

### Beta-galactosidase staining on mouse fetal liver section

Day 13.5 mouse embryos from WT and *Arhgef3* KO were harvested and whole embryo fixation and beta-galactosidase staining was carried out according to the manual protocol from the Cellular Senescence Assay kit (Millipore, Billerica, MA, USA). After beta-galactosidase staining, whole embryos were mounted in tissue TeK (Sakura Finetek, USA) and stored at -80°C. 7μm frozen sections including liver tissue, were mounted onto lysine coated slides (Sigma, St. Louis, MO, USA) and allowed to air dry. Immunohistochemistry was carried out using a primary rat-anti mouse CD41 (BD, USA) and a secondary biotinylated goat anti-rat IgG antibody, and developed using the 3,3’diaminobenzidine (DAB) substrate kit for peroxidase (Vector laboratories, Burlingame, CA, USA). Nuclei were counterstained with nuclear fast red (Sigma, St. Louis, MO, USA) and Digital images captured using an Axiocam (Zeiss, Thornwood, NY, USA) camera mounted onto an Axioskop fluorescent microscope (Zeiss).

### Isolation and culture of murine bone marrow cells

Hips, femurs, and tibias of 4–6 week-old mice were dissected and isolated. Bone marrow was obtained after crushing in ice-cold PBS containing 1% fetal bovine serum (FBS). Following centrifugation, red blood cell lysis (BD Pharmlyse, San Jose, CA) was performed. Next, cells were washed with PBS containing 2 mM EDTA and further cultured or stained for flow cytometry analysis. For MK differentiation, bone marrow cells were grown at 5x10^6^ cells/ml in StemSpan (StemCell Technologies, Vancouver, Canada) supplemented with 30% BIT9500 (StemCell Technologies) plus 20 ng/ml murine TPO for a total of 4 days before flow cytometry analysis.

### Flow cytometric analysis of DNA content and surface markers

Fresh bone marrow cells or *in vitro* differentiated MK were stained with FITC-conjugated anti-CD41 antibody, then fixed and permeabilized using 70% Ethanol at 4°C overnight. After incubation with 4 ug/ml RNAase at 37°C for 4 hr, nuclear DNA was stained with 1g/ml propidium iodide (Company) and analyzed using a FACS Calibur cytometer (BD Biosciences, San Jose, CA) and FlowJo software (TreeStar, Ashland, OR).

### Determination of bleeding times

Using a sharp razor blade, 0.5 cm of the tail was removed and the tail held in warm PBS. Time until cessation of tail bleeding was measured. Persistent bleeding was terminated after 5 minutes.

### Murine platelet preparation and analysis

For platelet counts, peripheral blood was collected from the retro-orbital sinus using EDTA coated tubes, and assessed using a Hemavet Analyzer (Drew Scientific, FL). For functional platelet assays, blood was collected into heparinized tubes (BD Biosciences, San Jose, CA). Platelet-rich plasma was prepared as previously described (REF). For flow cytometry, 50 uL of whole blood was added to 200 uL Tris-buffered saline (20mM Tris-HCl, 137mM NaCl). After further dilution with 1 mL of 2 mM CaCl2 in modified Tyrodes-HEPES buffer (5mM HEPES, 140mM NaCl, 2.7mM KCl, 5.5mM dextrose, 0.42mM Na2HPO4, 12mM NaHCO3), platelets were stained with anti-GPIIb/IIIa (CD41/61) FITC together with anti-GPIIb/IIIa (CD41/61) PE (JONA clone) or anti-P-selectin PE (Emfret, Würzburg, Germany). During staining, platelets were also stimulated with 1mM adenosine 5-diphosphate (ADP; Sigma-Aldrich, St. Louis, MO, USA), 0.1U or 0.01U Thrombin (Roche, Basel, Switzerland), or 3mM Thromboxane A2 analog U46619 (Cayman Chemical, Ann Arbor, MI, USA) at 37°C for 20min. Reactions were stopped by adding 300 ul 0.5% PFA (in PBS) to the samples and analyzed using a FACS Calibur cytometer (BD Biosciences, San Jose, CA, USA) and FlowJo software (TreeStar, Ashland, OR, USA).

### Transient immunothrombocytopenia model

To induce transient thrombocytopenia, 4-week-old mice were intravenously injected with 2 μg/g body weight of anti-GPIb antibody (R300; Emfret, Würzburg, Germany). Platelet counts were then assessed every other day for 10 days with the Hemavet Analyzer (Drew Scientific, Texas, USA).

### Human megakaryocyte differentiation

CD34+ progenitor cells were isolated and enriched from cord blood as reported previously.(51) MK cultures of 10^5^ cells/ml were supplemented with 100 ng/ml thrombopoietin (rhTPO CellGenix, Freiburg, Germany) and 10 ng/ml IL1b (R&D, Minneapolis, MN, USA) in CellGro media (CellGenix, Freiburg, Germany). Media and cytokines were rejuvenated at days 3 and 6. At day 10, mature MKs were immunopurified using an anti-CD42b PE conjugated antibody (Pab5, NHS Blood and Transplant, IBGRL, Bristol, England) and a PE positive selection kit (STEMCELL Technologies, Vancouver, Canada). MK purity was verified by FACS and shown to be >95% for CD41a (BD, San Jose, CA, USA) and CD42b (IBGRL, Bristol, England).

### Human platelet function association study

Healthy individuals (366 females and 501 males, of predominantly Northern European origin) were recruited in the two phases. First 500 in Platelet Function Cohort (PFC) 1 in 2007, and the remaining ones in 2010–2011. Of the 500 PFC-1 samples, 480 were genotyped using the genomewide platform Illumina 610K.[28] The PFC-2 samples were genotyped using the genome-wide platform, Illumina OmniExpress.

Functional response of platelets was measured for P-selectin expression and fibrinogen binding on platelets in response to activation with a mid-range dose of ADP and the cross-linked (XL) collagen mimetic collagen related peptide (CRP) using the protocol described by Jones et al. [29] The 67 sentinel SNPs (excluding HLA-B and HLA-DOA to reduce any spurious associations) that were previously found to be associated to either platelet count or volume [5] were tested for association with platelet function as described by Jones et al. [29] The association study was carried out using an additive model that corrected for batch effects of PFC1 and PFC2.

### Expression quantitative trait locus (eQTL) analysis

Genotyping for rs1354034 was obtained from 154 healthy human subjects of the Platelet RNA and eXpression study-1 (PRAX1) using the Illumina HumanOmni5 platform, as described previously. [30] ARHGEF3 expression levels on the same 154 PRAX1 subjects were profiled using the Affymetrix HuGeneST1.0 platform, as described previously. [11] Association between allele dosage of rs1354034 and ARHGEF3 expression levels was tested using simple and multiple linear regression models. In the simple model allele dosage and ARHGEF3 expression levels were used as the explanatory and dependent variables, respectively. In the multiple linear regression model, allele dosage was used to explain ARHGEF3 expression levels after accounting for the subjects’ age, gender and race.

### Statistical analysis

Statistical significance was assessed set at P<0.05 and using Prism 6.0 software (GraphPad Software) using the standard t-test statistical analysis.

### Human subjects

All research involving human participants was approved by the authors' Institutional Review Boards, and all clinical investigation was conducted according to the principles expressed in the Declaration of Helsinki. Written informed consent was obtained from the participants. Subjects participating in the Cambridge Platelet Function cohort study (Cambridge, UK) provided written signed consent under Research Ethics Committee (REC) reference number 05/Q0104/27 approved by Huntingdon Research Ethics Committee and Cambridgeshire Research Ethics Committee. The epigenome studies were executed as part of the BLUEPRINT consortium studies and subjects participating in the BLUEPRINT study (Cambridge, UK) provided their written signed consent under the Research Ethics Committee approved study with REC reference number 12/EE/0040 by the NRES Committee East of England, Hatfield. The TEMPLE targeted sequencing of the extended ARHGEF3 locus was performed with DNA samples from participants in the Lifelines Cohort Study in the Netherlands. These participants provided written signed consent under the Ethics Committee approved study with CCMO reference number NL17981.042.07.

## Supporting information

S1 Fig rs1354034 has different allele frequencies in the African American and Caucasian populations. (TIF)

S2 Fig *Arhgef3* KO mice have normal progenitor cell population in the bone marrow and normal MK differentiation. (A) Progenitor cell population analysis by flow cytometry in fresh isolated bone marrow cells from WT and *Arhgef3* KO mice showed no difference in progenitor population. (B) Megakaryocyte ploidy and maturation assessment from freshly isolated (left) or 4 day *in vitro* differentiation (right) bone marrow cells shows no defects of MK maturation in the *Arhgef3* KO.
(TIF)

S1 Table Raw data for data shown in S1 Fig. (PDF)
