# Supplementary figures and images for: SNP in human ARHGEF3 promoter is associated with DNase hypersensitivity, transcript level and platelet function, and Arhgef3 KO mice have increased mean platelet volume
Source: PLoS One. 2017 May 23;12(5):e0178095. doi: 10.1371/journal.pone.0178095 (PMC5441597; doi:10.1371/journal.pone.0178095)

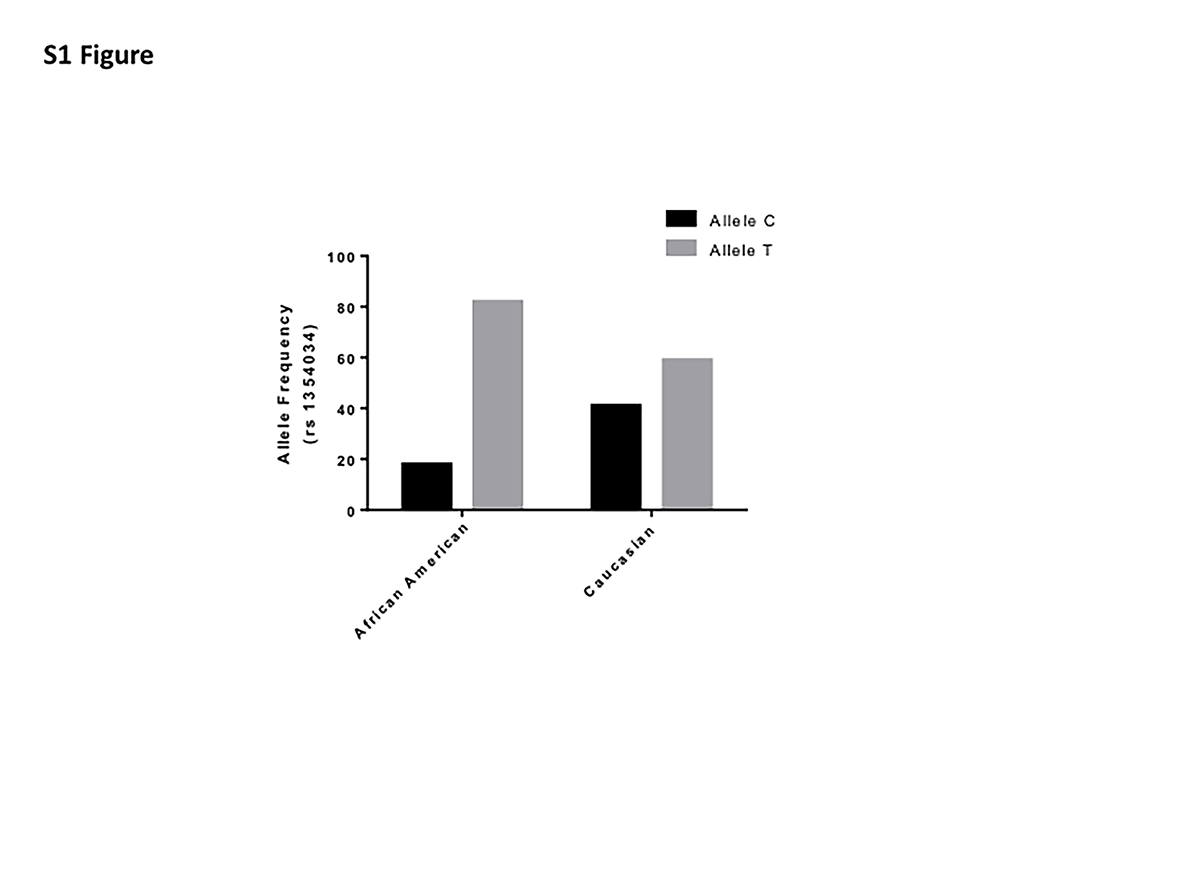

Supplement: S1 Fig — (TIF) [file pone.0178095.s001.tif]

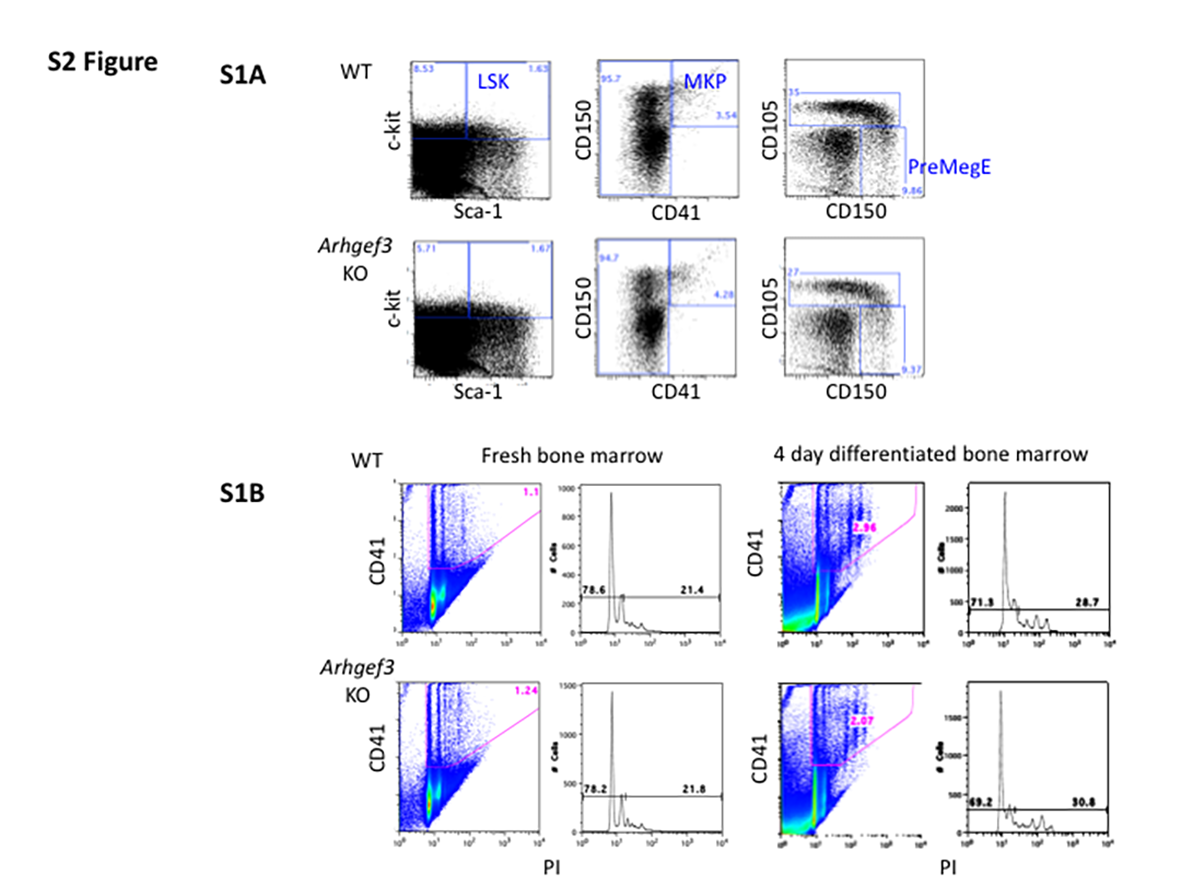

Supplement: S2 Fig — (A) Progenitor cell population analysis by flow cytometry in fresh isolated bone marrow cells from WT and Arhgef3 KO mice showed no difference in progenitor population. (B) Megakaryocyte ploidy and maturation assessment from freshly isolated (left) or 4 day in vitro differentiation (right) bone marrow cells shows no defects of MK maturation in the Arhgef3 KO. (TIF) [file pone.0178095.s002.tif]
