# Supplementary material for: SNP in human ARHGEF3 promoter is associated with DNase hypersensitivity, transcript level and platelet function, and Arhgef3 KO mice have increased mean platelet volume
Source: PLoS One. 2017 May 23;12(5):e0178095. doi: 10.1371/journal.pone.0178095 (PMC5441597; doi:10.1371/journal.pone.0178095)
Supplement: S1 Table — (PDF) [file pone.0178095.s003.pdf]

Supplemental Data

Primary eQTL data for rs1354034

ARHGEF3_expression	rs1354034
3.30973	CT
3.68549	CC
3.96534	CT
4.37309	CT
4.2915	TT
5.4892	TT
3.84676	CT
3.56324	CT
3.77577	CC
3.73943	CC
3.26124	CC
4.727	CT
3.66965	CC
4.62882	TT
4.88451	TT
4.48035	TT
3.88601	TT
3.99102	CC
4.52874	TT
3.49618	CT
3.8054	CT
3.88461	CT
4.51584	TT
3.99908	TT
4.59443	TT
3.69597	CT
3.22031	TT
4.54888	CT
3.56714	CC
4.11085	TT
3.05578	CC
4.10163	CT
5.03549	TT
3.32625	CT
3.7776	TT
3.99492	CT
4.02959	CT
4.01435	TT
5.08524	TT
4.20636	CT
3.07812	CC

3.46208	CC
3.91229	CC
3.25541	CC
3.3119	CC
3.98134	CT
4.86391	TT
3.80953	CC
3.4716	CT
4.70956	TT
3.28835	CC
4.06998	TT
4.30821	TT
3.47509	CT
4.22163	CT
4.14067	TT
4.31726	CT
3.35914	CC
3.82456	CT
3.21342	CC
4.09589	CC
4.76945	CT
3.3093	CC
3.86396	TT
4.11063	CT
3.62128	CT
4.08281	CC
3.75719	CT
3.84777	CT
4.27202	CT
3.95818	CT
3.44693	CT
3.91183	TT
4.53186	TT
3.37533	CT
3.85123	CC
3.76864	CT
3.68134	CC
4.88564	TT
4.32916	TT
4.57801	CT
3.22442	CT
3.4334	CT
3.43199	CT
3.64784	CT
4.64441	TT
3.74094	TT

3.45539	CC
4.39723	TT
4.93342	TT
2.9904	CC
3.69056	CC
4.35791	TT
3.77099	CC
3.55301	CT
4.62473	TT
3.89224	CC
3.9346	CT
4.62557	TT
3.24786	CC
3.83191	CT
4.25507	CC
3.41616	TT
3.0907	CC
4.5482	TT
3.83787	CC
3.69547	CC
3.70412	CT
4.34887	TT
3.54917	CT
3.71675	CC
3.85827	TT
4.4518	TT
3.93046	CT
3.93153	TT
3.65126	CC
3.58785	CC
3.9222	CT
3.65433	CT
3.46022	CC
3.03029	CC
4.1217	TT
2.99206	CC
4.94093	TT
3.82421	CT
4.34538	TT
5.13091	TT
3.93178	TT
4.17578	TT
4.03381	CT
3.27334	TT
4.21157	TT
3.73911	CT

4.38247	CT
4.24745	CT
3.64479	TT
4.0268	CT
3.85173	TT
3.85221	CT
4.15207	CT
4.06376	CT
3.99817	TT
4.10561	CT
3.84349	CT
4.18306	CT
4.20249	TT
4.02575	CT
4.18307	CT
4.16771	CT
4.36279	TT
4.41669	CT
3.54113	TT
3.63556	CT
3.74207	CC
